# Transcriptomics of Biostimulation of Plants Under Abiotic Stress

**DOI:** 10.3389/fgene.2021.583888

**Published:** 2021-02-03

**Authors:** Susana González-Morales, Susana Solís-Gaona, Marin Virgilio Valdés-Caballero, Antonio Juárez-Maldonado, Araceli Loredo-Treviño, Adalberto Benavides-Mendoza

**Affiliations:** ^1^CONACYT-Universidad Autónoma Agraria Antonio Narro, Saltillo, Mexico; ^2^UPL, Saltillo, Mexico; ^3^Departamento de Botánica, Universidad Autónoma Agraria Antonio Narro, Saltillo, Mexico; ^4^Departamento de Alimentos, Universidad Autónoma de Coahuila, Saltillo, Mexico; ^5^Departamento de Horticultura, Universidad Autónoma Agraria Antonio Narro, Saltillo, Mexico

**Keywords:** gene expression, PGPRs, macroalgae, peptides, humic acid, chitosan, selenium, silicon

## Abstract

Plant biostimulants are compounds, living microorganisms, or their constituent parts that alter plant development programs. The impact of biostimulants is manifested in several ways: via morphological, physiological, biochemical, epigenomic, proteomic, and transcriptomic changes. For each of these, a response and alteration occur, and these alterations in turn improve metabolic and adaptive performance in the environment. Many studies have been conducted on the effects of different biotic and abiotic stimulants on plants, including many crop species. However, as far as we know, there are no reviews available that describe the impact of biostimulants for a specific field such as transcriptomics, which is the objective of this review. For the commercial registration process of products for agricultural use, it is necessary to distinguish the specific impact of biostimulants from that of other legal categories of products used in agriculture, such as fertilizers and plant hormones. For the chemical or biological classification of biostimulants, the classification is seen as a complex issue, given the great diversity of compounds and organisms that cause biostimulation. However, with an approach focused on the impact on a particular field such as transcriptomics, it is perhaps possible to obtain a criterion that allows biostimulants to be grouped considering their effects on living systems, as well as the overlap of the impact on metabolism, physiology, and morphology occurring between fertilizers, hormones, and biostimulants.

## Introduction

Biostimulation has been described as a general biological phenomenon dependent on the interactions between the cell’s molecular structures and the external physical, chemical and biological stimuli, or impulses. The biostimulation results in the alteration of metabolic processes that allows the most efficient use of environmental resources, substantially increased growth or yield, and increased tolerance to adverse environmental factors (Juárez-Maldonado et al., 2019). It would be expected that starting from the definition of biostimulation that the definition of biostimulant would be derived; but in reality, that of biostimulant preceded that of biostimulation as a result of the need for regulation in the agricultural sector, which has found a very relevant niche in biostimulants (du Jardin, 2015).

Indeed, at present, biostimulants have gained importance in agriculture from ecological and commercial perspectives. Biostimulants are labeled as ecologically benign since most are considered biodegradable, non-toxic, non-polluting, and non-hazardous for various organisms (Yakhin et al., 2017).

Currently, there is no agreed-upon legal or academic definition of biostimulant; thus, there are several definitions of this term. One academic definition of a plant biostimulant refers to any substance or microorganism applied to plants to improve nutrient use efficiency, stress tolerance, and/or quality traits of plants, regardless of their nutrient content (du Jardin, 2015). Another definition defines biostimulants as materials that, when applied in small amounts, are capable of promoting growth in plants (Juárez-Maldonado et al., 2019).

It should be noted that, from the biological point of view, biostimulants are all those biotic and abiotic factors that induce biostimulation (including physical factors such as UV radiation, physicochemical factors such as nanomaterials, and biological factors such as pests, pathogens, competitors, and beneficial organisms and microorganisms) (Juárez-Maldonado et al., 2019). However, for purposes of developing this review with a focus in agriculture, we start from the biostimulants definition and categorization developed by du Jardin (2015). In his paper, the author classified biostimulants into seven groups: (1) beneficial bacteria, (2) beneficial fungi, (3) algae and botanical extracts, (4) protein hydrolysates and other nitrogen-containing compounds, (5) humic acid and fulvic acid, (6) chitosan and other biopolymers, and (7) inorganic compounds.

Currently, the advancement of omics sciences through the development of genome sequencing technologies, as well as the reduction in the costs of these techniques, have revolutionized the understanding of the identification of metabolic pathways in plants (Owen et al., 2017). The review is based on the supposition that through transcriptomic studies, it is possible to identify molecular markers (Harper et al., 2016) that are associated with common responses in a wide range of crop species; these molecular markers can, in turn, be used to understand the mode of action of biostimulants, their interaction with the environment, and the genotype of plants (De Palma et al., 2019). The present review contributes to the description, in terms of transcriptomics, of the impact of the different categories of biostimulants on agricultural crop species, with an emphasis on corn, rice, wheat, tomato, and *Arabidopsis*. It is intended to describe the transcriptomic landscape induced by biostimulants in plants under abiotic stress. Abiotic stress, such as salinity, drought or temperature variation, can decrease productivity and cause considerable losses in crop yields by more than 60% (Singhal et al., 2016). The following sections are included in this manuscript: (1) beneficial bacteria; (2) beneficial fungi; (3) algae and botanical extracts; (4) protein hydrolysates and other nitrogen-containing compounds; (5) humic acid and fulvic acid; (6) chitosan and other biopolymers; and (7) inorganic compounds. In each section, we present works that describe the genes that whose transcript level changes in plants in response to the application of a biostimulant (in a specific category) in plants under abiotic stress. In total, 56 recent works (published from 2010 to the present) from the seven biostimulant category groups were included.

## Beneficial Bacteria

Microorganisms are widely used to produce biostimulants (Xavier and Boyetchko, 2002; Sofo et al., 2014; Colla et al., 2015; Matyjaszczyk, 2015). Biostimulants based on microorganisms are preparations that include living and/or non-living microorganisms and their metabolites. Within this category, the most studied microorganisms are those defined as plant growth-promoting bacteria (PGPBs) (Calvo et al., 2014). Among the PGPBs, the genres most studied for use as biostimulants include *Bacillus* (Adesemoye et al., 2010), *Azospirillum*, *Pseudomonas*, *Streptomyces*, *Achromobacter*, and *Rhizobium* (Calvo et al., 2014). The plant-promoting effects of PGPBs are mostly explained by the release of metabolites directly stimulating growth. The direct mechanism includes those in which the bacteria produce growth regulators [auxins, gibberellins, cytokinins, ethylene, and abscisic acid (ABA)] that are ultimately incorporated into the plant system and thus affect the balance of plant growth regulators, or they act as a sink for plant-released hormones that induce changes in plant metabolism, promoting the overall growth of the plant (Govindasamy et al., 2010). The indirect mechanisms include biological nitrogen fixation, phosphate solubilization, production of siderophores, antibiotics, and other metabolites, production of defense enzymes and modulation of plant stress markers, induction of systemic resistance (ISR) and competition within the rhizosphere (Kumar et al., 2020).

Genes and their function are described below using the results of 10 studies in which beneficial bacteria-based biostimulants were applied to plants under abiotic stress (Table 1).

**TABLE 1:** Description of genes whose transcript level changed in response to the application of bacteria-based biostimulants in plants under abiotic stress.

**Gene/gene expression time**	**Biostimulant type**	**Abiotic stress type**	**Crop/gene expression organ**	**References**
*COX1*, *PKDP*, *bZIP1*, *AP2-EREBP*, *Hsp20*, *COC1*/65 days old	*Pseudomonas fluorescens*	Drought	Rice/leaves	Saakre et al., 2017
*ACS*, *AC*/45 days after stress	*Ochrobactrum pseudogrignonense*, *Pseudomonas* sp., and *Bacillus subtilis*	Drought	*Vigna mungo* and *Pisum sativum* L./leaves and roots	Saikia et al., 2018
*OsNAM*, *OsGRAM*/not specified	*Bacillus amyloliquefaciens*	Salinity	Rice/roots	Chauhan et al., 2019
*UP1*, *NAS*, *MT*, *MTR-1-P*, *VITs*, *P450*, *HRP*, *UP2*/not specified	*Arthrobacter nitroguajacolicus*	Salinity	Wheat/roots	Safdarian et al., 2019
*RD29A*, *RD29B*, *RD20*, *RD22*, *KIN1*, *ERD1*/not specified	*Bacillus oryzicola*	Salinity	*Arabidopsis/*shoots and roots	Baek et al., 2020
*IA24*, *IA41*, *IA19*/after 6 h at heat stress, after 6 h at cold stress, after 5 days without water	*Bacillus amyloliquefaciens* subsp. *plantarum*	Heat, cold, drought	Wheat/leaves	Abd El-Daim et al., 2018
*APX*, *CAT*, *SOD*, *RBCS*, *RBCL*, *H*^+^*-PPase*, *HKT1*, *NHX1*, *NCED*/24 days after transplantation	*Serratia liquefaciens*	Salinity	Maize/leaves	El-Esawi et al., 2018b
*P5CS1*, *SOD*, *APX1*, *CAT*, *SHSP*/45 days from sowing	*Bacillus cereus*, *Providencia rettgeri* and *Myroides odoratimimus*	Chromium and heat stresses	Sorghum/leaves	Bruno et al., 2020
*CAPIP2*, *CaKR1*, *CaOSM1*, *CAChi2*/7 days after inoculation	*Bacillus fortis*	Salinity	Capsicum/leaves	Yasin et al., 2018
*APX1*, *CAT1*, *SOD2*, *SOD4*, *APX2*, *PR1*, *PRP2*, *PRP4*, *HSP70*/32 days after transplant	*Azospirillum brasilense* and *Rhizobium tropici*	Salinity	Maize/leaves and roots	Fukami et al., 2018

The effect of a biostimulant from *Pseudomonas fluorescens* applied to reproductive-stage rice plants under drought stress (water restriction for 15 days) was analyzed (Saakre et al., 2017), and six differentially expressed genes (DEGs), *COX1*, *PKDP*, *bZIP1*, *AP2-EREBP*, *Hsp20*, and *COC1*, were reported in leaves of 65-day-old plants. Differentially expressed transcripts correspond to the cytochrome oxidase subunit 1 (*COX1*) gene, protein kinase domain protein (*PKDP*), bZIP protein family (*bZIP1*) members, APETALA2-ethylene-responsive element-binding protein (*AP2-EREBP*), heat-shock protein 20 (*Hsp20*), and the circadian oscillator component (*COC1*). *bZIP1*, *AP2-EREBP*, *COC1*, and *PKDP* genes are considered regulatory proteins. *bZIP1*, *AP2-EREBPs*, and *COC1* genes are considered stress-induced transcription factors (TFs) involved in the ABA-dependent signaling pathway (Reeves et al., 2011; Seung et al., 2012). ABA is generally defined as a stress hormone since it intervenes in the regulation of the response to biotic and abiotic stress (Vishwakarma et al., 2017); it also plays a vital role in physiological processes such as seed germination, dormancy, and stomatal closure (Akpinar et al., 2012). In the ABA-dependent signaling pathway, protein receptors (PYR/PIL/RCAR) bind to ABA by inhibiting PP2C activity, allowing SnRK2 activation by autophosphorylation. Subsequently, active SnRK2 can phosphorylate TFs such as AREB/ABF, DREB2A/2B, RD22BP1, and MYC/MYB TFs, which bind to their corresponding cis-elements and modulate the expression of response genes (de Zelicourt et al., 2016; Bulgakov and Wu, 2019). The *PKDP* gene encodes an enzyme with kinase activity that participates in phosphorylation reactions; this enzyme is a key component of signal transduction (Mohanta et al., 2015) and is involved in multiple cellular processes in plants such as growth and development, carbon and nitrogen metabolism, the formation of the cytoskeleton, senescence and cell death, hormone signal transduction, regulation of ion channels, and the defense response (Lei et al., 2007). *Hsp20* and *COX1* genes are considered functional proteins. The *Hsp20* gene encodes a small (12–42 kDa) heat-shock protein (sHsp) with chaperone activity; its function is to degrade denatured proteins, and it has an ATP-dependent chaperonin function. These proteins can bind partially folded or denatured proteins and avoid permanent unfolding or aggregation (Reddy et al., 2015; Nagaraju et al., 2020). The *COX1* gene encodes an enzyme with cytochrome oxidase activity, which is related to the process of apoptosis, a type of programmed cell death (PCD) in plants. PCD is triggered in plants during biotic and abiotic stress since recycling and mobilizing nutrients are carried out under adverse conditions; this process can be triggered by reactive oxygen species (ROS) (Foyer and Noctor, 2005). Moreover, autophagy (another type of PCD) is related to protein turnover and removal of damaged organelle proteins, which can lead to changes in cell behavior (Akpinar et al., 2012).

As a strategy to improve *Vigna mungo* L. and *Pisum sativum* L. plants under conditions of water deficit (polyethylene glycol-6000), (Saikia et al., 2018) a PGPB consortium (*Ochrobactrum pseudogrignonense*, *Pseudomonas* spp., and *Bacillus subtilis*) was applied. The plants treated with the consortium presented, in addition to increased growth, a better adaptation to resist stress due to downregulation of the *ACS* and *ACO* genes at 45 days after stress. In the plants that had only stress, the opposite occurred, and the expression of these genes was increased. The *ACS* gene encodes the ACC synthase enzyme, and the *ACO* gene encodes the ACC oxidase enzyme. ACC synthase is responsible for producing ACC, and ACC oxidase is involved in the production of ethylene (Li et al., 2020b). Ethylene is a hormone related to plant growth and development as well as processes such as fruit ripening and abscission, leaf senescence, seed germination, and organogenesis (Trujillo-Moya and Gisbert, 2012); however, a sudden increase in ethylene concentration in the plant during stress causes negative effects and leads to senescence (Czarny et al., 2006). The mechanism by which the expression of the *ACS* and *ACO* genes is reduced is triggered because the consortium of bacteria are producers of hormones, specifically indoleacetic acid (IAA), which triggers the production of relatively high concentrations of ACC and, subsequently, feedback inhibition of IAA synthesis (Glick, 2012). Additionally, some rhizobial strains produce the enzyme ACC deaminase, which removes some of the ACC (the immediate precursor to ethylene) before it can be converted to ethylene (Ma et al., 2002).

In rice plants under salinity stress (100 mM NaCl), the biostimulant effect of *Bacillus amyloliquefaciens*-SN13 was evaluated (Chauhan et al., 2019). During stress, *B. amyloliquefaciens* increased the relative water content, biomass, proline content and total soluble sugar content in plants while decreasing the electrolyte leakage and lipid peroxidation. Alterations in gene expression were also observed in the transcriptome of rice roots under salt stress with *B. amyloliquefaciens.* Changes in the expression of a considerable number of genes related to the stress response, hormones, photosynthesis, lipid metabolism and cell wall were induced. Additionally, DEGs in functional categories such as the stress response (*Os11g26750*, *Os10g38600*, *Os05g28740*, and *Os05g45480*), metabolism (*Os03g08860*, *Os04g27060*, *Os01g43750*, *Os05g41460*, *Os04g47360*, and *Os03g12510*), transporters (*Os07g15460*), and regulation (*Os04g56990*, *Os06g41770*, *Os09g39650*, *Os09g31031*, *Os03g14590*, *Os11g03370*, and *Os05g10620*) were quantified. The functional validation in *Saccharomyces cerevisiae* of the *OsNAM* and *OsGRAM* genes with 5- and 3-fold induction, respectively, in transcriptome of rice roots with *B. amyloliquefaciens* under salinity was analyzed. Transformed yeast cells expressing *OsNAM* and *OsGRAM* showed enhanced tolerance under osmotic stress; moreover, the transformed cells were able to survive at relatively high temperatures, and they displayed increase tolerance to arsenite and arsenate. *OsNAM* belongs to the NAC family of TFs, which is a large plant-specific gene family that is related to the regulation of tissue development and the response to abiotic stress (Shan et al., 2020). *OsGRAM* is important for the ABA response, hormone signaling under stress conditions, and the perception of environmental stimuli and regulation of the response to those stimuli (Mauri et al., 2016).

The effect of *Arthrobacter nitroguajacolicus* (seeds inoculated with 10^8^ CFU mL^–1^) on wheat tolerance to salinity stress (200 mM NaCl) has been studied (Safdarian et al., 2019). Transcriptomic analysis revealed upregulation of 152 genes, and 5 genes were significantly downregulated. Inoculated roots of plants under salt stress presented differential expression of many genes involved in stilbenoids, diarylheptanoid metabolism, phenylpropanoids, flavonoids, terpenoid, porphyrin, and chlorophyll metabolism. RNA-seq results, 11 DEGs (including *UP1*, *NAS*, *MT*, *MTR-1-P*, *VITs*, *P450*, *UP2*) were quantified. *UP1* is a functional cis-element responsible for germination-associated gene expression (Tatematsu et al., 2008). *NAS* is induced by salt stress and is related to niacinamide, the synthetic precursor of plant iron (Johnson et al., 2011). *MT* gene encodes a metal chelatin protein involved in the detoxification of heavy metals and in the homeostasis of intracellular metal ions (Ahn et al., 2012). *MTR-1-P* encodes a 5-methylthioribose 1-phosphate related to ethylene biosynthesis (Arraes et al., 2015). VIT proteins are involved in the transport and storage of iron, can maintain iron within the optimal physiological range and prevent cellular toxicity (Cao, 2019). Cytochrome P450 proteins are related to signals for growth and development, are responsible for protecting plants from different stresses, and are involved in in redox reactions and a large number of biosynthetic pathways (Bak et al., 2011; Safdarian et al., 2019). *UP2* encodes an uncharacterized conserved protein and is related to cell structure (Duan et al., 2017).

In *Arabidopsis thaliana* plants under salt stress (80 and 100 mM NaCl), the biostimulant effect of *Bacillus oryzicola* was studied (Baek et al., 2020). Compared with control plants, plants with *B. oryzicola* while under salinity showed increased numbers of lateral roots, increased fresh weight, and increased chlorophyll content, and they accumulated less salt-induced malondialdehyde and Na^+^. Additionally, plants with *B. oryzicola* while under salinity presented enhanced transcription of the *RD29A*, *RD29B*, *RD20*, *RD22*, *KIN1*, and *ERD1* genes in the shoots and roots. The *RD29A* (which is responsive to desiccation) gene encodes a protein strongly related to the *Brassicaceae* family. Its function is similar to that of LEA proteins (Kaur and Gupta, 2005). *RD29B* encodes a protein that is induced in response to water deprivation, low temperature, salinity, and desiccation, and its response is mediated by ABA (Bihmidine et al., 2013). *RD20* is a stress-inducible gene that belongs to the caleosin family and is dependent on ABA signaling. *RD20* plays a role in drought tolerance through stomatal control under water-deficit conditions (Aubert et al., 2010). The *RD22* (which is responsive to desiccation) gene can be induced by stress caused by drought and salinity and by exogenous applications of ABA, but its role in the stress response is unknown (Wang et al., 2012). The *KIN1* gene encodes a protein induced in response to cold, dehydration, osmotica and ABA, possibly functioning as an antifreeze protein (Wang et al., 1995). The *ERD1* (early responsive to dehydration stress 1) protein functions in chloroplasts by degrading unassembled or misfolded proteins (Peltier et al., 2001).

Using wheat plants under heat (6 h at 45°C), cold (6 h at −5°C) and drought stress (5 days without water), the effect of *B. amyloliquefaciens* subsp. *plantarum* was studied (Abd El-Daim et al., 2018). cDNA-AFLP analysis revealed differential expression of more than 200 transcript-derived fragments (TDFs) in wheat leaves. Five TDFs (*IA20*, *IA24*, *IA35*, *IA41*, *IA19*) were selected for RT-PCR analysis. All the TDFs changed in expression pattern in plants treated with *B. amyloliquefaciens* subsp. *plantarum* and with the different stresses. *IA19*, *IA20*, and *IA24* are ABA responsive homologous. *IA20* encodes to TF WRKY20, this TF enhance drought tolerance regulating ABA signaling (Luo et al., 2013).

The effect of *Serratia liquefaciens* on maize plants under salinity stress (0, 80, and 160 mM NaCl) was studied (El-Esawi et al., 2018b). *S. liquefaciens* inoculation significantly reduced oxidative stress markers but increased the maize growth and biomass production along with better antioxidant defense system, leaf gas exchange, osmoregulation, and nutrient uptake under salinity. Also, it was found the upregulation of stress-related genes (*APX*, *CAT*, *SOD*, *RBCS*, *RBCL*, *H^+^-PPase*, *HKT1*, *NHX1*) and downregulation *NCED* gene. The *APX* gene encodes ascorbate peroxidase, which catalyzes the reduction of H_2_O_2_ and the oxidation of ascorbate, generating monodehydroascorbate (Eltelib et al., 2012). The *CAT* gene encodes catalase, and this enzyme is important in the removal of H_2_O_2_ generated in peroxisomes by oxidases involved in the β-oxidation of fatty acids, photorespiration and purine catabolism (Gill and Tuteja, 2010). The *SOD* gene encodes an enzyme that is crucial in the first line of antioxidant defense since it can convert highly reactive O_2_^–^ radicals into H_2_O_2_ and O_2_ in response to oxidative stress (Wang W. et al., 2016). The *RBCS* gene encodes the small Rubisco subunit and is part of a multigenic family. The Rubisco enzyme catalyzes the assimilation of CO_2_ in plants and is related to the photosynthesis process (Atkinson et al., 2017). *RBCL* is involved in photosynthesis, encodes the large subunit of the primary CO_2_ fixation enzyme Rubisco. This enzyme serves as the primary engine of carbon assimilation being the most abundant protein on earth (Igamberdiev, 2015). The *NCED3* (nine-cis-epoxycarotenoid dioxygenase 3) gene is related to the pathways of ABA biosynthesis and is highly expressed at the root level (Li et al., 2020a). *H^+^-PPase* ecodes H^+^-pumping pyrophosphate and *HKT1* encodes high-affinity K^+^ transporter 1 related with ion balance regulators. *NHX1* encodes Na^+^/H^+^ antiporter, which participates in Na^+^ sequestration, export and recirculation (Chen et al., 2016). Using sorghum plants under chromium (200 mg K_2_Cr_2_O_7_ kg^–1^ in soil) and heat stress (42°C during day and 28°C at night), the effect of *Bacillus cereus*, *Providencia rettgeri*, and *Myroides odoratimimus* (chromium reducing-thermotolerant CRT-PGPB) was studied (Bruno et al., 2020). Inoculation with CRT-PGPB increased plant growth, antioxidant status and decreased malondialdehyde and proline contents in plants under stress. Also, gene expression studies down-regulated the expression of *P5CS1* gene, and up-regulated the expression of *SOD*, *APX1*, *CAT* and *SHSP*. All genes were already mentioned.

The effect of *Bacillus fortis* (halotolerant HPGPR) on capsicum plants under salt stress (1 and 2 g NaCl kg^–1^ soil) was studied (Yasin et al., 2018). HPGPR promote growth attributes, chlorophyll, protein content and water use efficiency on capsicum plants under salinity. Also, up-regulated the expression profiles of stress related genes including *CAPIP2*, CaKR1, CaOSM1, and CAChi2. *CAPIP2* encodes plasma membrane intrinsic protein related in transportation of smaller neutral solutes and water (already mentioned). *CaKR1* is a member of ankyrin repeat zinc finger protein TF family, the over-expressed of this TF exhibited enhanced antioxidant metabolism (Seong et al., 2007). *CaOSM1* is a osmotin gene related with resistance against biotic and abiotic stress (Choi et al., 2013). *CAChi2* encodes chitinase class II is related with osmotic stress tolerance (Hong and Hwang, 2006).

Using maize plants under salinity stress (170 mM NaCl), the effect of *Azospirillum brasilense* and *Rhizobium tropici* inoculation was studied (Fukami et al., 2018). Inoculation affected antioxidant enzymes, proline, and MDA contents in leaves and roots. The expression of genes related to antioxidant activity were up-regulated (*APX1*, *CAT1*, *SOD2*, *SOD4* in leaves, and *APX2* in roots), while the expression of pathogenesis-related genes *PR1*, *prp2*, *prp4i* and *hsp70* were down-regulated in leaves and roots. Antioxidant genes and were already mentioned. PR1 (a member of a multigene family) is a salicylic acid inducible marker gene for systemic acquired resistance (SAR) (Hussain et al., 2018). The gene *prp2* encodes β-1, 3-glucanase, and *prp4* encodes chitinase family (Fukami et al., 2017). The gene *hsp70* were already mentioned.

Examination of Table 1 allows determining the absence of transcriptomic responses that were common among the 10 studies. One possible explanation, in addition to the small number of studies available, is that the interactions between the plant–bacteria are specific. There were no analogous responses between the different species studied. If to the above it is added that not all the studies were carried out under the same type of stress, it can be concluded that the possibility of demonstrating that a certain class of biostimulant can induce common responses in plants depends on carrying out studies with several species of plants treated with the same type of biostimulant under the same stress condition. However, common genes in some studies were related to ABA, TFs, and antioxidants.

## Beneficial Fungi

This category includes fungi and arbuscular mycorrhizal fungi (AMFs), which are considered microbial inoculants along with bacteria (Dodd and Ruiz-Lozano, 2012). Among AMFs, those of the *Glomus* genus have been widely studied (Calvo et al., 2014). Fungi of the genera *Neotyphodium*, *Curvularia*, *Colletotrichum*, *Fusarium*, *Alternaria*, and *Trichoderma* have been studied as biostimulants in plants, with *Trichoderma* being the most studied (Calvo et al., 2014; Jardin, 2015; De Palma et al., 2019).

Genes and their functions are described below using the results of eight studies in which beneficial fungi and AMFs were applied to plants under abiotic stress (Table 2).

**TABLE 2:** Description of genes whose transcript level changed in response to the application of fungi and AMF-based biostimulants based to plants under abiotic stress.

**Gene/gene expression time**	**Biostimulant type**	**Abiotic stress type**	**Crop/gene expression organ**	**References**
*LsPIP1*/60 days after transplant	*Glomus intraradices* (AMF)	Salinity	Lettuce/roots	Jahromi et al., 2008
*MIOX1*, *GLX1*, *CSD1*, *TT5*/after 0, 1, 3, and 6 days of stress	*Glomus mosseae* (AMF)	Drought	*Poncirus trifoliata/*leaves	Fan and Liu, 2011
*SOD*, *POD*, *CAT*/15 days of NaCl treatment	*Trichoderma longibrachiatum* (fungi)	Salinity	Wheat/leaves	Zhang et al., 2016
*MDHAR1*, *MDHAR2*, *DHARc*, *DHARp*, *GRc*, *GRp*, *Fe-SODp*, *Cu/Zn-SODp*, *APXc*/3 days after 50% of seeds germinated	*Trichoderma harzianum* (fungi)	Drought	Tomato/shoots and roots	Mastouri et al., 2012
*RBCL*, *PPH*, *Cu-Zn SOD*, *CAT*, *APX*, *GR*/6 days after stress	*Funneliformis mosseae* (AMF)	Salinity-alkalinity	Watermelon/leaves	Ye et al., 2019
*LePT1*, *LePT2*, *LePT3*, *LePT4*, *LePT5*/not specified	*F. mosseae* and *Rhizophagus intraradices* (AMF)	Drought	Tomato/roots	Volpe et al., 2018
*XM_020345004.1*, *XM_020296045.1*, *AY973229.1*/8 weeks after transplanting germinated seeds	*F. mosseae* (AMF)	Drought	Wheat/roots	Moradi Tarnabi et al., 2020
*23660*, *26140*, *20188*, *18003*, *4379*, *20176*, *6553*, *23129*, *29567*/10 days after stress	*Rhizophagus irregularis* (AMF)	Salinity	Asparagus/leaves	Zhang et al., 2019

In lettuce plants (Jahromi et al., 2008), researchers studied the effect of applying *Glomus intraradices* AMFs under salinity stress at two levels (50 and 100 mM NaCl). Compared with plants without the application of AMFs while with stress, plants treated with *G. intraradices* while under salinity stress presented increased growth and an increase in the relative water content. Additionally, the expression of the gene of an intrinsic plasma membrane protein (*LsPIP1*) increased 60 days after transplanting; *LsPIP1* encodes a type of aquaporin in the plasma membrane and is related to the transport of water and small solutes with no charge (Wang et al., 2019).

*Trichoderma longibrachiatum* inoculated (10^8^ CFU mL^–1^) onto wheat seedlings under salt stress (150 mM NaCl) (Zhang et al., 2016) can enhance the plant stress tolerance. The relative water content in the leaves and roots, the chlorophyll content, the proline content and the root activity were increased, but the content of leaf malondialdehyde under salinity stress was decreased. The antioxidant enzymes SOD, POD, and CAT were increased, and the relative expression of the *SOD*, *POD*, and *CAT* genes was upregulated. The possible mechanisms by which salinity suppresses the negative effect on wheat may be due to the improvement of the antioxidant defense system. *SOD* and *CAT* are discussed above. The *POD* gene encodes a peroxidase with a variety of biological functions, including hydrogen peroxide detoxification, hormone signaling, lignin biosynthesis and stress responses (Gao et al., 2010).

In tomato seedlings under water deficit, inoculation of a *Trichoderma harzianum* biostimulant was evaluated (Mastouri et al., 2012). The enhanced redox state of inoculated plants could be explained by the increased activity of antioxidant enzymes. Additionally, *T. harzianum* modulated the expression of genes encoding antioxidant enzymes. The *MDHAR1*, *MDHAR2*, *DHARc*, *DHARp*, *GRc*, *GRp*, *Fe-SODp*, *Cu/Zn-SODp*, and *APXc* genes showed increased expression in the shoots, whereas only the *APXc* gene showed increased expression in the roots. The *MDHAR* gene encodes a monodehydroascorbate reductase, and together with *DHAR* (dehydroascorbate reductase) and *GR* (glutathione reductase), it participates in the ascorbate-glutathione cycle. This cycle plays an important role in the efficient removal of excess ROS. *APX* and *SOD* are discussed above.

The effect of *Glomus mosseae* (AMF) inoculation on the drought (3 days of water depletion) tolerance of *Poncirus trifoliata* seedlings was studied (Fan and Liu, 2011). Plants inoculated with *G. mosseae* showed an increase in growth and increased relative water and chlorophyll contents. Under drought, the inoculated plants showed increased levels of proline and increased activity of antioxidant enzymes (SOD, POD). Additionally, four genes (*MIOX1*, *GLX1*, *CSD1*, *TT5*) involved in ROS homeostasis and counteracting oxidative stress presented increased expression in inoculated plants. The *MIOX1* gene encodes myo-inositol oxygenase, which is involved in the biosynthesis of ascorbic acid; ascorbic acid has been shown to be an important antioxidant protecting plants against oxidative damage (Fan and Liu, 2011). The *GLX1* gene encodes glyoxalase I, a key enzyme involved in the glutathione-based detoxification of methylglyoxal, a product of lipid and carbohydrate metabolism. The *CSD1* gene encodes a copper/zinc SOD, and this gene is discussed above. *TT5* (transparent testa 5) encodes a chalcone isomerase involved in flavonoid synthesis. Flavonoids have an important role in the modulation of ROS levels (Fan and Liu, 2011).

Using watermelon plants under salinity-alkalinity stresses (irrigation with 400 mL 60 mM salinity-alkalinity solution [NaCl, Na_2_SO_4_, NaHCO_3_, Na_2_CO_3_]), the effect of *Funneliformis mosseae* (AMF) was studied (Ye et al., 2019). The photosynthesis related parameters were alleviated after incubation of AMF. Under stress, the relative expression level of *RBCL*, *Cu-Zn SOD*, *CAT*, *APX*, *GR* were increased after AMF treatment. *PPH* was reduced after AMF treatment. RBCL, *Cu-Zn SOD*, *CAT*, *APX*, *GR* genes are involved in antioxidant metabolism and are discussed above.

The effect of *F. mosseae* and *R. intraradices* (AMF) on tomato plants under water stress (leaf water potential of about −0.9 and −1.0 MPa) was studied (Volpe et al., 2018). Gene expression analysis involved in inorganic phosphate uptake and transport was made. *LePT1*, *LePT2*, *LePT3*, *LePT4*, and *LePT4* changed their expression under water deficit and AMF treatment. P uptake, transfer and delivery are improved in AM roots. P fertilization can increase stress tolerance and productivity in several plant species (Sawers et al., 2017).

Using wheat plants under water deficit stress (each pot three times weekly irrigating, 25 ml per time), the effect of *F. mosseae* (AMF) was studied (Moradi Tarnabi et al., 2020). The results showed that symbiotic association between plant and AMF and irrigation not only affect transcription profile of the plant growth, but also membrane components and cell wall. The most DEGs were observed in lipid and carbohydrate metabolic process, membrane transports, cellulose synthase activity, chitinase activity, and nitrogen compound metabolic process related genes. The expression of three randomly selected DEGs were examined. The selected genes were associated to chitinase activity (*AY973229.1*), cellulose biosynthetic process (*XM_020345004.1*), and beta-glucosidase BoGH3B-like (*XM_020296045.1*). Chitin-related genes such as chitinase detect chitin molecules of fungus as a signal to trigger a defense response an increase the plant tolerance (Moradi Tarnabi et al., 2020). A stress-response related role can be considered for cellulose, since cellulose microfibrils and the other factor that lead the direction of cell growth can be regulated by water availability (Wang T. et al., 2016). Beta-glucosidase have important in cell wall biogenesis, which strongly provides protection against biotic and abiotic stress (Moradi Tarnabi et al., 2020).

The effect of *Rhizophagus irregularis* (AMF) on asparagus plants under salinity stress (100 mM NaCl) was studied (Zhang et al., 2019). The authors conducted a transcriptome analysis on leaves of garden asparagus to identify gene expression under salinity stress. 455 DEGs were identified in plants with salinity to plants with AMF and salinity. The expression profiles of 9 DEGs (23660, 26140, 20188, 18003, 4379, 20176, 6553, 23129, 29567) were by qRT-PCR. Their putative functions involved in nitrogen metabolism, synthesis of secondary metabolites, ion homeostasis, osmotic adjustment, and scavenging ROS, among others.

The review of Table 2 again, as in Table 1, reveals a small number of studies available. Except for the expression of antioxidant enzymes, where there were analogous responses between the different species studied, more studies are needed with the same species of beneficial fungus in different species of plants, under the same stress condition, to effectively demonstrate that the determination of the transcriptomic landscape will contribute to the omic definition of biostimulant.

## Algal and Botanical Extracts

Recently, marine algal extracts have been used as biostimulants, as their use in agriculture had previously been limited to being a fertilizer or a source of organic matter (Jardin, 2015). Marine macroalgae are divided into three broad groups (brown, red, and green), of which brown algae are the most widely used in agriculture; brown algae include species such as *Fucus* spp., *Sargassum* spp., *Laminaria* spp., *Turbinaria* spp., and *Ascophyllum nodosum*, this last of which is the most studied (Blunden and Gordon, 1986; Ugarte et al., 2006; Hong et al., 2007). The biostimulant function of algal extracts is commonly associated with the content of hormones such as cytokinins, auxins, abscisic acid, gibberellins, and other classes of hormone-like compounds such as sterols and polyamines (Craigie, 2011; Wally et al., 2013). However, they also contain compounds such as polysaccharides (laminarin), alginates, carrageenans, macro- and micronutrients, and nitrogenous compounds such as betaines (Khan et al., 2009; Craigie, 2011).

In the case of botanical extracts, substances extracted from plants (seeds, leaves, roots, and exudates) of various families have generally been used in agriculture as pesticides; in terms their functions as biostimulants, there is little research, thus representing an important area of opportunity (Ertani et al., 2013; Ziosi et al., 2013; Yakhin et al., 2017).

Genes and their functions are described below for 14 studies where biostimulants based on extracts of algae and botanicals were applied to plants under abiotic stress (Table 3).

**TABLE 3:** Description of genes whose transcript level changed in response to the application of biostimulants based on extracts of algae and botanicals to plants under abiotic stress.

**Gene/gene expression time**	**Biostimulant type**	**Abiotic stress type**	**Crop/gene expression organ**	**References**
*AtDREB2a*, *AtRD29*, *AtNFYA1*, *AtNFYA2*, *AtUBC24*, *AtWAK2*, *AtSYG1*, *At3g27150*/6 and 12 h after treatment	Extract of *A. nodosum*	Salinity	*Arabidopsis*/whole plant	Shukla et al., 2018
*LEA3*, *LEA1*, *CCA1*, *HVA22d*, *LTP6*, *DREB1A*, *DREB1C*, *LEA2*, *VA22b*, *Di21*, *SnRK2*, *CIPK25*, *LTP*, *RING*, *MYB*/1 and 5 days after treatment	Extract of *A. nodosum*	Salinity	*Arabidopsis*/whole plant	Jithesh et al., 2018
*ANN1*, *ANN2*, *PIP1*, *P5CS1*, *CHS*, *APX1*, *GPX3*/not specified	Extract of *A. nodosum* and 5-aminolevulinic acid (foliar application)	Salinity	*Asparagus aethiopicus*/leaves	Al-Ghamdi and Elansary, 2018
*COR15A*, *RD29A*, *CBF3*/when plants were exposed at −2°C	Extract of *Ascophyllum nodosum*	Cold	*Arabidopsis/*leaves	Rayirath et al., 2009
*P5CS1*, *P5CS2*, *PRODH*/not specified	*A. nodosum* lipophilic components	Freezing	*Arabidopsis*/leaves	Nair et al., 2012
*NCED3*, *MYB60*, *RAB18*, *RD29A*, *RbCS1A*, *RCA*, *PIP1;2*, *B ca1*, *PsbS*, *VDE*, *DFR*, *APX2*, *SOD*/From the moment of stress until the fourth day	Extract of *A. nodosum*	Drought	*Arabidopsis/*leaves	Santaniello et al., 2017
*GmCYP707A1a*, *GmCYP707A3b*, *GmRD22*, *GmDREB1B*, *GmERD1*, *FIB1a*, *GmPIP1b*, *GmGST*, *GmBIP*, *GmTp55*/75 h and 59 h after treatment	Extract of *A. nodosum*	Drought	Soybean/leaves	Shukla et al., 2017
*TAS14*/7th day of stress	Extract of *A. nodosum* (foliar application)	Drought	Tomato/leaves	Goñi et al., 2018
*TaNCED3.1*, *TaNCED3.2*, *TaHAI1*, *DREB family*, *TaRap2-4*, *TaMYB31*, *TaAREB3*, *dehydrin*, *TaP5CS1*, *TaNCED4*, *TaNCED2*, *TaERD2*/6 days after stress	Extract of *Gracilaria dura*	Drought	Wheat/leaves	Sharma et al., 2019
*GRMZM2G439784*, *GRMZM2G324221*, *GRMZM2G164129*, *GRMZM2G163866*/10 days after treatment	Extract of *Kappaphycus alvarezii*	Drought	Maize/roots	Kumar et al., 2019
*SOD*, *CAT*, *APX*, *DAHR*, *GR*, *PrxQ*/not specified	Licorice root extract	Salinity	Pea (*Pisum sativum* L.)/leaves	Desoky et al., 2019
*DtDREB2A*, *DtMYB30*, *DtNAC019*, *DtNAC72*, *DtNAC19*, *DtNAC69*, *DtZIP63*, *DtABF3*, *DtHB12*, *DtHB7*/2, 4, 6, 9, and 24 h from exposure to stress	Extract of borage	Salinity	Wild rocket/leaves	Franzoni et al., 2019
*CuZnSOD*, *MnSOD*, *CAT*, *FeSOD*/24 and 48 h after stress	Lignin derivatives, plant-derived aminoacids and molybdenum	Heat	Cucumber/seeds	Campobenedetto et al., 2020
*ObOLP*/3-months old plants	*Moringa oleifera* extract	Salinity	*Ocimum basilicum/*leaves	Alkuwayti et al., 2020

In *A. thaliana* plants under freezing stress (−2°C), the effect of extracts (lipophilic fraction) of the brown macroalga species *A*. *nodosum* (1 g L^–1^) was studied (Rayirath et al., 2009), where the expression of three cold-responsive genes (*CBF3*, *RD29A*, and *COR15A*) increased when the plants were at −2°C. The authors pointed out that in response to the application of the extract of *A. nodosum* to seedlings of *A. thaliana*, freezing tolerance was obtained by protecting the integrity of the membrane; also, compared with the untreated seedlings, the treated seedlings presented a smaller decrease in the concentration of chlorophyll as a result of stress. The *CBF3* (C-repeat-binding factor) gene is a TF that plays an important role in tolerance to low temperatures in *Arabidopsis* (Takuhara et al., 2011). *CBF3* is activated in response to low temperatures and dehydration and is independent of ABA; *CBF3* binds to the cis-element DRE-CRT (C-repeat/dehydration-responsive element) that is present in the promoter region of the *RD29A* and *COR15A* genes in *Arabidopsis*, the *WCS120* gene in wheat and the *BN115* gene in *Brassica napus* (Medina et al., 2011). Moreover, there are indications that *CBF3* regulation is carried out through responses to light quality and the circadian rhythm (Fowler et al., 2005; Franklin and Whitelam, 2007). The *RD29A* gene is discussed above. The *COR15A* (cold-regulated) gene regulates cold tolerance by stabilizing chloroplast membranes (Thalhammer et al., 2014).

In *A*. *thaliana* plants to which lipophilic components of an extract of *A*. *nodosum* (ANE), the effect of tolerance to stress by freezing (−2°C for 24 h) was studied (Nair et al., 2012). Expression of the *P5CS1*, *P5CS2*, and *ProDH* genes was detected; the expression of *P5CS1* and *P5CS2* increased, while that of *ProDH* decreased. These genes are involved in proline synthesis (*P5CS1* and *P5CS2*) and degradation (*ProDH*). *P5CS1* and *P5CS2* encode delta1-pyrroline-5-carboxylate synthase enzymes that regulates the rate of proline biosynthesis. This gene is expressed in some tissues under normal conditions and throughout the plant under conditions of water deficit, in addition to being induced by ABA and salt stress (Kesari et al., 2012). *ProDH* catalyzes the degradation of proline to produce glutamic acid; this gene is related to decreasing the oxidative burst and cell death associated with the hypersensitive response (Cecchini et al., 2011).

In *A*. *thaliana* plants under drought stress (absence of a hydroponic solution for 4 days), the effect of an extract of *A*. *nodosum* was evaluated (Santaniello et al., 2017); the extract positively influenced the survival of the plants. The plants under stress and treated with the extract presented increased expression of the *NCED3*, *MYB60*, *RAB18*, *RD29A*, *RbCS1A*, *RCA*, *PIP1;2*, β*CA1*, *PsbS*, *VDE*, *DFR*, *APX2*, and *SOD* genes 4 days after stress. These genes are involved in the pathways of the antioxidant system and are ABA dependent. The *MYB60* gene is related to the regulation of stomatal movement, and the expression of this gene increases with low levels of ABA; additionally, in an initial drought state, this gene can induce root growth. In contrast, in a severe state of drought, its expression is inhibited, resulting in stomatal closure and a decrease in root growth (Oh et al., 2011). The *RAB18* gene (which is sensitive to ABA) encodes a glycine-rich hydrophilic protein (Hoque et al., 2012) that belongs to the LEA family of proteins and has a dehydrin function (Hernández-Sánchez et al., 2019). *RAB18* protects membranes under dehydration conditions by binding to anionic phospholipids through electrostatic forces (Eriksson and Harryson, 2011), in addition to binding to other proteins to prevent their denaturation (Graether and Boddington, 2014). The *RD29A* gene is discussed above. Similarly, the RCA gene encodes the Rubisco activase enzyme, which is a chloroplastic enzyme encoded in the nucleus and participates in the activation of Rubisco (Hasse et al., 2015); this gene is also related to jasmonate-induced leaf senescence (Elizabete Carmo-Silva and Salvucci, 2013). Two genes related to the regulation of mesophilic diffusion restriction include *PIP1;2* (discussed above) and β*CA1* (β-carbonic anhydrase 1), both of which participate in carboxylation or decarboxylation reactions related to photosynthesis and respiration; as such, they play an important role in the catalysis of CO_2_ and water to form protons and bicarbonate (Hu et al., 2015). The *PsbS* (photosystem II subunit S) and *VDE* (violaxanthin depoxidase) genes are involved in the photoprotection mechanism to avoid damage caused by oxidative stress in plants due to excess energy from sunlight (Fufezan et al., 2012; Ciszak et al., 2015). The *DFR* (dihydroflavonol reductase) gene is involved in the biosynthesis of anthocyanins, which protect plants from stress through their activity of ROS detoxification (Cui et al., 2014). The *APX2* and *SOD* genes are discussed above.

Researchers (Shukla et al., 2017) studied the effect of an extract of *A. nodosum* on soybean plants under drought stress (without irrigation). Compared with the untreated plants, the treated plants had higher relative water content, antioxidant activity, and stomatal conductance under drought stress. In addition, there were changes in the expression of the *GmCYP707A1a*, *GmCYP707A3b*, *GmRD22*, *GmDREB1B*, *GmERD1*, *GmNFYA3*, *FIB1a*, *GmPIP1b*, *GmGST*, *GmBIP*, and *GmTp55* genes at 75 h (stress) and 89 h (recovery) after treatment. The *GmCYP707A1a* and *GmCYP707A3b* genes encode ABA 8′-hydroxylases, which participate in the regulation of ABA levels during dehydration and rehydration (Umezawa et al., 2006; Zheng et al., 2012a). *GmRD22* is discussed above. The *GmDREB1B* (dehydration response element-binding) gene belongs to a family of TFs induced by drought and salinity, and this gene is ABA dependent. This TF binds to the cis-element DRE-CRT, which is present in the promoter of the *COR* and *RD29A* genes, both of which are related to the abiotic stress response (Tuteja, 2007). *GmERD1* is discussed above. The *FIB1a* gene improves the phototolerance of photosystem II (PSII) (Mutava et al., 2015), and the *GmPIP1b* gene is also discussed above. The *GmGST* gene, via its glutathione reduction potential, detoxifies ROS and protects cells from oxidative damage (Mcgonigle et al., 2000). The *GmBIP* gene encodes a chaperone protein that is related to delayed senescence in leaves and therefore increases tolerance to drought stress (Carvalho et al., 2014). The *GmTp55* gene encodes an aldehyde dehydrogenase enzyme that reduces reactive aldehydes derived from lipid peroxidation under oxidative stress (Wang et al., 2017).

Using tomato plants under drought stress, researchers (Goñi et al., 2018) studied the effect of several commercial products based on extracts of *A*. *nosodum*. All of the ANEs affected drought stress tolerance but to different degrees. Regulation of the *TAS14* gene was reported 7 days after stress, and this gene was differentially overexpressed in response to applications of all extracts. *TAS14* encodes a group 2 LEA protein called dehydrin, which is induced by osmotic stress and ABA (Godoy et al., 1994). When this gene is overexpressed, long-term tolerance to drought and salinity is achieved through the reduction in osmotic potential and the accumulation of sugars and potassium (Muñoz-Mayor et al., 2012).

Using wheat plants under water stress (no water for 10 days), researchers (Sharma et al., 2019) evaluated foliar applications of *Gracilaria dura* (red algae) sap. The expression levels of the *TaNCED3.1*, *TaNCED3.2*, *TaHAI1*, *DREB*, *TaRap2-4*, *TaMYB31*, *TaAREB3*, *dehydrin*, *TaP5CS1*, *TaNCED4*, *TaNCED2*, and *TaCla013224* genes were analyzed, which increased on the sixth day of the onset of stress. *TaNCED3.1*, *TaNCED3.2*, *TaNCED4*, and *TaNCED2* belong to the *NCED* family of genes, which encode 9-cis-epoxycarotenoid dioxygenases; these are key enzymes involved in ABA biosynthesis and are regulated in response to drought and salinity (Behnam et al., 2013). *TaHAI1* encodes a member of the PP2C family, whose members includes class A and type 2C phosphatase proteins and are related to the downregulation of osmotic stress and ABA signaling (Nguyen et al., 2019). *DREBs* are discussed above. *TaRap2-4* encodes a DREB-subfamily TF related to light mediation and ethylene signaling (Lin et al., 2008). *TaMYB31* belongs to a subfamily of TFs, is ABA dependent and plays an important role in the development of and defense response in plants (Yanhui et al., 2006; Tuteja, 2007). *TaAREB3* is also a TF that is sensitive to ABA and is related to stomatal movement and ROS generation in response to ABA (Wang et al., 2013). *Dehydrins* are LEA-like proteins (as mentioned above), and *TaP5CS1* participates in proline biosynthesis (as mentioned above). *TaERD2* encodes an HSP70-type chaperonin that is synthesized in response to stress and is the main chaperone in maintaining protein homeostasis (Rowarth et al., 2019).

The effect of *Kappaphycus alvarezii* extract applied to the soil was analyzed on maize plants under drought stress (no irrigation for 10 days) (Kumar et al., 2019). To obtain a global view of the effect, the authors analyzed the transcriptome of the roots of the plants 10 days after the application of the treatment. A total of 896 upregulated genes and 533 downregulated genes were differentially expressed. However, only 4 genes were overexpressed in response to the application of the extract and stress compared to the application of the extract only, and 18 genes were repressed. The overexpressed genes included *GRMZM2G439784*, *GRMZM2G324221*, *GRMZM2G164129*, and *GRMZM2G163866*. The *GRMZM2G439784* gene encodes an LRR-type kinase, which belong to the receptor kinase subfamily, and its function lies in communication between cells to transmit signals during development and before environmental stimuli to activate defense; it is of the utmost importance in resistance against pathogens (Antolín-Llovera et al., 2014; Dievart et al., 2015). *GRMZM2G324221* encodes a structural protein of the small ribosomal subunit (40S) and participates in the regulation of response to virus infections (Li, 2019). The function of *GRMZM2G164129* has not been characterized, whereas *GRMZM2G163866* is a high-affinity nitrate transporter; overexpression of the latter could translate into an improvement in nitrogen metabolism (Liu X. et al., 2014).

Using *A*. *thaliana* plants under saline conditions (150 mM NaCl), researchers (Shukla et al., 2018) studied the effects of an extract of *A*. *nodosum* by measuring the expression of several microRNAs (miRNAs) and their target genes. The miRNA *miR396a-5p* was downregulated, which repressed the expression of the *AtGRF7* gene. In turn, this repression positively regulated the expression of *AtDREB2a* and *AtRD29*; the greater expression of the *AtDREB2a* and *AtRD29* genes resulted in tolerance to salt stress. *AtDREB2a* and *AtRD29* are discussed above. The expression of the miRNA *ath-miR169g-5P* also increased, and as a result, the expression of the target nuclear factors *AtNFYA1* and *AtNFYA2* increased. *AtNFYA1* is associated with hypersensitivity to salt stress and ABA during the early stages of post-germination growth (Li et al., 2013). *AtNFYA2* participates in nitrogen metabolism, regulation of light signaling, and chloroplast biogenesis (Laloum et al., 2013; Petroni et al., 2013). The expression of the miRNAs *ath-miR399*, ath-*miR827*, and *ath-miR2111b* as well as their target genes *AtUBC24*, *AtWAK2*, *AtSYG1*, and *At3g27150* was also altered, suggesting a role of the extract of *A*. *nodosum* in phosphate homeostasis (Liu et al., 2012; Shukla et al., 2018).

Using *A*. *thaliana* plants under salinity stress (150 mM NaCl), researchers (Jithesh et al., 2018) studied the effect of extracts of *A*. *nodosum* subfractionated with ethyl acetate. The transcriptome of the plants was analyzed under salinity and in response to the application of the extract on the first and fifth days after salinity. On the first day, the expression of the *LEA3*, *CCA1*, *LEA1*, *HVA22d*, *LTP6*, *ATGOLS3*, *ATGOLS2*, *DREB1A*, and *DREB1C* genes increased. The products of *LEA* genes belong to a family of hydrophilic proteins with a protective function of functional proteins (Tunnacliffe and Wise, 2007) to prevent their aggregation (Chakrabortee et al., 2007). The *LEA3* gene is induced by drought and salinity (Du et al., 2016), and *LEA1* is induced by wounds and mild stress (Dunaeva and Adamska, 2001). *CCA1* has a regulatory function in the circadian rhythm and controls various processes, such as the stomatal opening (Hassidim et al., 2017). *HVA22d* is induced by ABA and by stress and is related to the regulation of autophagy (Chen et al., 2002, 2009). *LTP6* is predicted to encode a protein related to pathogenesis (PR) (Le et al., 2014). *ATGOLS3* and *ATGOLS2* encode the enzyme galactinol synthase, which is key to the biosynthesis of the oligosaccharides of the raffinose family and are related to tolerance to drought, salinity, and cold stress (Taji et al., 2002; Nishizawa et al., 2008). *DREB1A* and *DREB1C* are TFs of the DREB family, which is discussed above. On the fifth day after salinity, the expression of the *LEA1*, *LEA2*, *VA22b*, *Di21*, *SnRK2*, *CIPK25*, *LTP*, *RING*, and *MYB* genes increased. *VA22b* and *Di21* regulate stress and are induced by ABA (Jithesh et al., 2018). *SnRK2* is discussed above. *CIPK25* is a serine-threonine protein kinase that interacts with calcineurin (a calcium sensor); both of these are related to signal transduction in response to environmental stress (Kanwar et al., 2014). *LTP* is discussed above. *RING* and *MYBs* are TFs; *RING* acts as a ligase and participates in the regulation of gene expression in response to environmental or hormone signals (Qin et al., 2008), and *MYBs* are discussed above.

Using *Asparagus aethiopicus* plants subjected to salinity stress (2,000 and 4,000 mg L^–1^ NaCl), researchers (Al-Ghamdi and Elansary, 2018) studied the synergistic effects of the application of a commercial product based on *A*. *nodosum* and 5-aminolevulinic acid (ALA) applied via foliar treatment. ALA is a precursor of porphyrins, so it has effects on the photosynthetic apparatus, thus stimulating the defense system in plants (Wu et al., 2018). The application of both products caused a synergistic effect on plant growth associated with increased expression of the *ANN1*, *ANN2*, and *PIP1* genes, which are associated with the transport of water and Ca^+2^; the *P5CS1* and *CHS* genes, which are related to the production of secondary metabolites; and the *APX1* and *GPX3* genes, which are associated with antioxidant metabolism in plants. *ANN1* and *ANN2* encode a family of proteins called annexins, which are Ca^+2^ transporter permeases attached to the plasma membrane. They participate in processes such as stomatal closure, adaptation to stress, and cell signaling (Laohavisit et al., 2012; Wang J. et al., 2018). *PIP1* and *P5CS1* are discussed above. *CHS* encodes a chalcone synthase, which is a key enzyme involved in flavonoid biosynthesis and is also involved in auxin transport (Brown et al., 2001; Dana et al., 2006). *APX1* and *GPX3* play a role in antioxidant metabolism, and *GPX3* acts as a redox transducer whose function is similar to that of *APX1* in H_2_O_2_ homeostasis and is related to ABA signal transduction during stress (Miao et al., 2006).

Using pea plants under salinity stress (150 mM NaCl for 2 weeks), researchers (Desoky et al., 2019) studied the effect of applying licorice (*Glycyrrhiza glabra*) root extract on pea seeds. Saline stress reduced seedling growth and increased oxidative stress; however, in pretreated seedlings, mitigation of these effects was observed. Treatment with the extract also increased the transcription of the *CAT*, *SOD*, *APX*, *GR*, *DHAR*, and *PrxQ* genes, decreasing oxidative stress. *CAT*, *SOD*, *APX*, *GR*, *DHAR*, and *PrxQ* (peroxiredoxin) participate in the cellular antioxidant system, which maintains ROS homeostasis to mitigate oxidative damage. However, ROS are essential for maintaining metabolic flow and activating acclimation responses to stress through systemic signaling (Ahmad et al., 2010; Suzuki et al., 2012).

Using wild rocket (*Diplotaxis tenuifolia* L.) plants under salinity stress (200 mM NaCl), researchers (Franzoni et al., 2019) studied the effect of a foliar application of a borage (*Borago officinalis*) extract. The expression of several TFs related to salinity stress was studied at 2, 4, 6, 9, and 24 h after exposure to stress. TFs such as *DtDREB2A*, *DtMYB30*, *DtNAC019*, *DtNAC72*, *DtNAC19*, *DtNAC69*, *DtZIP63*, *DtABF3*, *DtHB12*, and *DtHB7* presented positive regulation. *DtDREB2A* is discussed above. *DtMYB30* is an ABA-sensitive TF that participates in processes such as germination and response to stress (Zheng et al., 2012b). *DtNAC019*, *DtNAC72*, *DtNAC19*, and *DtNAC69* compose a family of TFs related to development and stress responses in plants, respond to ABA, and promote the antioxidant system (Xu et al., 2015). *DtZIP63* is a TF that regulates the circadian cycle through a low-energy response and is activated by a kinase (SnRK1) (Frank et al., 2018). *DtABF3* is a TF induced by ABA and osmotic stress (Bogamuwa and Jang, 2014), and *DtHB12* and *DtHB7* are ABA-dependent TFs and act by mediating the growth response to water stress (Olsson et al., 2004).

Using cucumber plants (*Cucumis sativus* L.) subjected to heat stress (35°C), researchers (Campobenedetto et al., 2020) studied the effect of a seed application of a biostimulant based on lignin derivatives and containing plant-derived amino acids and molybdenum (KIEM^®^). The application of the biostimulant increased the percent germination, fresh biomass, and increased in expression levels *RBOHD*, *CuZnSOD*, *MnSOD*, *CAT*, and *GST* genes, while *FeSOD* gene was decreased. *CuZnSOD*, *MnSOD*, *CAT*, *GST*, and *FeSOD* are ROS-scavenging enzymes and are discussed above. *RBOHD* is a ROS-producing enzyme, H_2_O_2_ is a relatively long-lived ROS, and its accumulation is caused by the induction of membrane-bound respiratory burst oxidase homolog proteins (Rboh), which are important players in abiotic stress responses (Huang et al., 2014).

The effect of *Moringa oleifera* leaves extract applied on *Ocimum basilicum* plants under salt stress (1,000 mg L^–1^) was studied (Alkuwayti et al., 2020). The application of *M. oleifera* extract altered the expression of *ObOLP* gene and was positively correlated with the plant growth and yield enhancement. *ObOLP* is a osmotin-like proteins, members of the pathogenesis-related protein 5 (PR-5), which are produced in plants under different abiotic and biotic stresses (Mayer Weber et al., 2014). Under salinity and drought stress, OLP maintains cellular osmolarity by compartmentalization of solutes or by structural and metabolic alterations (Chowdhury et al., 2017).

Examination of Table 3 allows determining a few of transcriptomic responses that were common among the 14 studies. Common genes in some studies were related to ABA, TFs, antioxidants, and LEA proteins. Most of the studies were carried out in *A. nodosum* in different types of stress. Regarding algae extracts, most of the studies were carried out on *A. nodosum* under different types of stress. However, in the case of botanical extracts, there are few related studies. More studies are needed with extracts of the same species of algal and botanical in different species of plants, under the same stress condition.

## Protein Hydrolysates and Other Nitrogen-Containing Compounds

The two main categories of protein-based products are divided into: (1) protein hydrolysates consisting of mixtures of peptides and amino acids that can be of animal or plant origin and (2) individual amino acids such as proline, glutamate, glutamine, and glycine betaine (Calvo et al., 2014). Mixtures of amino acids and peptides can be obtained by chemical, enzymatic, or thermal hydrolysis from byproducts of plant and animal origin (Calvo et al., 2014; Jardin, 2015). Chemical synthesis can be used to produce amino acids or mixtures of these. This category also includes other nitrogenous molecules, such as polyamines, betaines, and non-protein amino acids (Vranova et al., 2011), which are diverse in higher plants but are poorly characterized in terms of their physiological and ecological functions (Vranova et al., 2011).

Genes and their function are described below based on the results of seven studies in which biostimulants based on protein hydrolysates and other nitrogen-containing compounds were applied to plants under abiotic stress (Table 4).

**TABLE 4:** Description of genes whose transcript level changed in response to the application of biostimulants based on protein hydrolysates and other nitrogen-containing compounds to plants under abiotic stress.

**Gene/gene expression time**	**Biostimulant type**	**Abiotic stress type**	**Crop/gene expression organ**	**References**
*Solyc02g084840*, *Solyc03g025810*/3 to 11 days after application of treatment	A mixture of amino acids, proteins, vitamins, and betaines	Drought	Tomato/leaves	Petrozza et al., 2014a
*ZmPAL*/12 days after stress	Hydrolysate of alfalfa plants, triacontanol, and indole 3-acetic acid	Salinity	Maize/leaves	Ertani and Schiavon, 2013
*ZmNRT2.1*, *ZmNRT2.2*, *ZmNRT2.3*, *ZmNAR2.2*, *ZmNRT1.1*, *ZmNRT1b*, *ZmNRT*, *ZmSOD1A*, *ZmRbohA*, *ZmRbohC*/3 days after stress	Protein hydrolysate	Hypoxia, salinity, nutrient deficiency	Maize/roots	Trevisan et al., 2019
*BI-1*, *MAPK1*, *WRKY53*, *CAT1*, *GPX*, *SOD*, *ATG1*, *ATG2*, *ATG4*, *ATG6*, *ATG7*, *ATG8*, *ATG9*, *ATG10*, *ATG13*/not specified	Panchagavya	Salinity	Rice/leaves	Khan et al., 2018
*CAT*, *MnSOD*, *WRKY53*, *BI-1*/not specified	Panchagavya	Salinity	Rice/leaves	Khan et al., 2017
*Cla018095*, *Cla010664*, *Cla004567*, *Cla009820*, *Cla012125*, *Cla003187*, *Cla021039*, *Cla007826*, *Cla013224*, *Cla001877*, *Cla001590*, *Cla006037*/not specified	Melatonin	Vanadium toxicity	Watermelon/roots	Nawaz et al., 2018
*HEMA1*, *CHLH*, *POR*/10 days after treatment	5-Aminolevulinic acid	Salinity	Cucumber/roots	Wu et al., 2018

Using tomato plants under drought stress (no water after the development of the third or fourth leaf), researchers (Petrozza et al., 2014b) evaluated the foliar application of a commercial product based on a complex mixture of vitamins, amino acids, proteins, and betaines. The results indicated that, compared with the untreated plants, the plants treated with the product while under stress had higher growth and stress tolerance. Additionally, the expression of the *Solyc02g084840* and *Solyc03g025810* genes was studied for 14 days after treatment; these genes were expressed between days 3 and 11. The *Solyc02g084840* gene is an ortholog of the *Arabidopsis RAB18* gene that is discussed above. *Solyc03g025810* is an ortholog of the *RD29B* gene, which encodes a protein that is induced in response to water deprivation, low temperature, salinity, and desiccation, and its response is mediated by ABA (Bihmidine et al., 2013).

Using maize plants under salinity stress (25, 75, and 150 mM NaCl), researchers (Ertani and Schiavon, 2013) evaluated a biostimulant based on a hydrolysate of alfalfa (*Medicago sativa* L.) plants, triacontanol (TRIA), and indole-3-acetic acid (IAA) added to the irrigation water for 48 h. Compared with untreated plants under stress, the treated plants under stress had greater biomass and presented greater activity of enzymes related to nitrogen metabolism. Plants treated with the biostimulant while under one of 3 levels of salinity stress presented increased expression of the *ZmPAL* gene at 12 days after stress. The *ZmPAL* gene encodes the enzyme phenylalanine ammonium lyase (PAL), which is a key enzyme that catalyzes the first step of the phenylpropanoid pathway, producing precursors of a wide variety of vital secondary metabolites related to plant defense, such as lignin, flavonoids, isoflavonoids, coumarin, and stilbenes (Huang et al., 2010).

Using maize plants treated with a commercial biostimulant based on a protein hydrolysate added to the hydroponic solution, researchers (Trevisan et al., 2019) evaluated the tolerance to three types of abiotic stress: hypoxia (deprivation of air bubbles in the liquid hydroponic solution), salinity (25 mM NaCl), nutrient deficiency (only distilled water was supplied in the hydroponic solution) and the combination of these. The treated plants had increased root and shoot growth and increased tolerance to single and combined stress conditions. Additionally, genes related to nitrate transport (*ZmNRT2.1*, *ZmNRT2.2*, *ZmNRT2.3*, *ZmNAR2.2*, *ZmNRT1.1*, *ZmNRT1b*, and *ZmNRT*) and ROS metabolism (*ZmSOD1A*) were expressed. The *ZmNRT2.1*, *ZmNRT2.2*, *ZmNRT2.3*, and *ZmNAR2.2* genes belong to the high-affinity nitrate transport system, and the *ZmNRT1.1*, *ZmNRT1b*, *ZmNRT* genes belong to the low-affinity nitrate transport system. An increase in the expression of these genes occurs since the application of protein hydrolysates in plants can modulate the expression of critical genes involved in the assimilation of nitrogen (transporters) (Sestili et al., 2018). *ZmSOD1A* is discussed above.

Using two rice cultivars (one susceptible and one tolerant to salinity) under salinity stress (100 mM NaCl), researchers (Khan et al., 2018) studied the effects of a natural biostimulant called panchagavya (a mixture of milk, butter, curd, urine, and cow dung) applied as a soil drench. The results indicated that the treated plants under salinity stress showed an improvement in the physiological and biochemical characteristics and presented increased expression of several genes: the expression of *BI-1*, *MAPK1*, and *WRKY53* increased in the tolerant variety; the expression of *CAT1*, *GPX*, and *SOD* increased in the susceptible variety; and the expression of *ATG1*, *ATG2*, *ATG4*, *ATG6*, *ATG7*, *ATG8*, *ATG9*, *ATG10*, and *ATG13* increased in both varieties. The *BI-1* gene is expressed during senescence and under various stress conditions, and its expression gradually decreases throughout the cell death process (Ishikawa et al., 2015). *MAPK1* belongs to a family of kinases (mitogen-activated protein kinases) that regulate the response to abiotic and biotic stress via signaling cascades (Neupane et al., 2019). *WRKY53* is an early response factor to water deficit; its expression regulates the stomatal response (Sun and Yu, 2015). The *CAT1*, *GPX*, and *SOD* genes are discussed above. The *ATG1*, *ATG2*, *ATG4*, *ATG6*, *ATG7*, *ATG8*, *ATG9*, *ATG10*, and *ATG13* genes are related to the autophagy process; together with PCD, this process regulates responses to stress since it is essential to degrade oxidized proteins during oxidative stress (Xiong et al., 2007).

The effect of the panchagavya biostimulant amendment to the soil drench applied to rice plants under salinity stress (100 mM NaCl) has been studied previously (Khan et al., 2017), and the results showed that stressed and treated plants presented upregulated *CAT*, *MnSOD*, *WRKY53*, and *BI-1* gene expression. All these genes are discussed above.

In watermelon plants under stress from exposure to vanadium (V) (50 mg L^–1^), the application of melatonin added to the irrigation solution was studied (Nawaz et al., 2018). The results indicated that treatment with melatonin reduced the concentrations of V in the leaves and stems and reduced the concentrations of H_2_O_2_ and malondialdehyde (MDA). The expression of the *Cla018095*, *Cla010664*, *Cla004567*, *Cla009820*, *Cla012125*, *Cla003187*, *Cla021039*, *Cla007826*, *Cla013224*, *Cla001877*, and *Cla001590* genes also increased. The *Cla018095* gene is related to chlorophyll biosynthesis. *Cla010664* encodes an O-methyl transferase, and *Cla004567* encodes an S-methyl transferase, which participates in the biosynthesis of melatonin. *Cla010664* can eliminate ROS or activate antioxidant enzymes such as SOD and CAT (Manchester et al., 2015). The *Cla009820* (superoxide dismutase), *Cla012125* (superoxide dismutase), *Cla003187* (peroxidase), *Cla021039* (glutathione peroxidase), *Cla013224* and *Cla007826* (glutathione S-transferase) genes encode enzymes with antioxidant activity. *Cla001877* is a respiratory burst oxidase and can integrate Ca^+2^ signaling and protein phosphorylation with ROS production, the last being key to the regulation of growth, development, responses to environmental stimuli, and cell death (Suzuki et al., 2011). *Cla001590* encodes a V-dependent haloperoxidase that may be related to the absorption of inorganic forms of iodine in plants, although its function has not been fully defined (Smoleñ et al., 2019).

Using cucumber plants under salinity stress (50 mmol L^–1^ NaCl), researches (Wu et al., 2018) evaluated a biostimulant based on ALA applied via foliar sprays. Plants exhibited increased photosynthesis (increased plant height and leaf area, increased gas exchange capacity, increased the use of light by photosystem II and improved chlorophyll biosynthesis) in response to the application of ALA and low stress conditions. There was an increase in the expression of the *HEMA1*, *CHLH*, and *POR* genes at 10 days after treatment. *HEMA1* encodes glutamyl-tRNA reductase, *CHLH* encodes Mg-chelatase, and *POR* encodes protochlorophyllide oxidoreductase. All three genes are involved in chlorophyll biosynthesis (Stephenson and Terry, 2008). As the chlorophyll content increased in response to the application of ALA, the tolerance of cucumber plants to stress also increased.

The review of Table 4 reveals a small number of studies available (7). Except for the expression of antioxidant enzymes, where there were analogous responses between the different species studied, more studies are needed with the same proteins, hydrolysates, and other nitrogen-containing compounds in different species of plants, under the same stress condition.

## Humic Acid and Fulvic Acid

Humic substances are natural components of soil organic matter resulting from the decomposition of animal, plant, and microbial waste (Jardin, 2015). They are considered the main components of soil organic matter and are the most abundant natural organic compounds on Earth (Calvo et al., 2014). Humic substances are heterogeneous compounds classified by their molecular weight and solubility in (1) humic acids, which are soluble in basic media, (2) fulvic acids, which are soluble in alkaline and acidic media, and (3) humins, which are not extractable from the soil (Berbara and García, 2014). In addition to their use as biostimulants, humic substances are related to key processes in the soil and plants, such as carbon and oxygen exchange between the soil and the atmosphere, availability of nutrients, and the detoxification and transport of toxic substances (Piccolo and Spiteller, 2003).

In this category, six studies related to humic and fulvic acid substances was found, where gene expression was analyzed (Table 5).

**TABLE 5:** Description of genes whose transcript level changed in response to the application of biostimulants based on humic acids and chitosan and other biopolymers to plants under abiotic stress.

**Gene/gene expression time**	**Biostimulant type**	**Abiotic stress type**	**Crop/gene expression organ**	**References**
*ZmPAL1*/48 h after treatment	Hummus of *Nicodrilus caliginosus*	None	Maize/leaves	Schiavon et al., 2010
*HSP101*, *HSP81.1*, *HSP26.5*, *HSP23.6*, *HSP17.6A*/9h after application of humic acid	Humic acids	Heat	*Arabidopsis*/not specified	Cha et al., 2020
*SnRK2.2*, *MDH*, *WRKY DNA-binding transcription factors family*/6 days after stress	Humic acids (vermicompost)	Weak acids	Maize/root	Baía et al., 2020
*SETIT_021707mg*, *SETIT_016840mg*, *SETIT_ 015030mg*, *SETIT_004913mg*, *SETIT_016654mg*/5 days after stress	Humic acid	Drought	Foxtail millet/leaves	Shen et al., 2020
*GME*, *AO*, *ALDH*, *GST*, *G6PDH*, *CYS-GYL*, *C4H*, *CHS*, *F3′5′H*, *F3H*/4 and 8 days under stress	Fulvic acid	Drought	*Camellia sinensis*/shoots	Sun et al., 2020
*CCoAOMT*, *CAB37*, *AGT1*, *At4g26520*/0, 4, 8, and 12 days after stress	Fulvic acid	Drought	*Paeonia ostii*/leaves	Fang et al., 2020
*SOD*, *JA*/48 h after treatment	nCu-chitosan-PVA and chitosan-PVA complex	Salinity	Tomato/leaves	Hernández-Hernández et al., 2018
*AOX1*/3 days after treatment	Chitosan	Salinity	Maize/leaves	Turk, 2019
*CAT3*/24 h after treatment	Xyloglucan oligosaccharides	Salinity	*Arabidopsis/*leaves	González-Pérez et al., 2018
*GH3*/24 h after treatment	Chitosan oligosaccharide	Cold	*Camellia sinensis/*leaves	Li et al., 2020e
*CAT*, *APX*, *POD*, *SOD*, *GmSALT3*, *CHS/*12 days of growth	Chitosan modified biochar	Salinity	Soybean/plant tissue	Mehmood et al., 2020

Using maize plants, researchers (Schiavon et al., 2010) evaluated a high-molecular-weight (>3,500 Da) humic fraction from *Nicodrilus caliginosus* feces, which was added for 48 h hydroponically. The effect on phenylpropanoid metabolism was subsequently evaluated, where the expression of the *ZmPAL1* gene increased considerably in plants treated with the humic fraction at three different concentrations. The *ZmPAL1* gene is discussed above.

Using *Arabidopsis* plants under heat stress (45°C), the application of humic acid (commercial product) was studied (Cha et al., 2020). The authors performed a transcriptomic analysis to identify the HA-prompted molecular mechanisms. Gene ontology analysis indicated that humic acid up-regulates diverse genes related in the response to stress. Heat stress causes induction in gene families such as heat-shock protein (HSP), coding genes including *HSP101*, *HSP81.1*, *HSP26.5*, *HSP23.6*, and *HSP17.6A*. HSPs function as molecular chaperones to protect against thermal denaturation of substrates and stimulate refolding of denatured substrates, also play an important role in maintaining cell membrane integrity, ROS scavenging and production of antioxidants, osmolytes (Khan and Shahwar, 2020).

Using maize seedlings under weak acids stress (acetic and salicylic acids), the application of humic acids extracted from vermicompost produced with cattle manure was studied (Baía et al., 2020). Humic acids decrease the intracellular pH and produce high level of *SnRK2.2* and *MDH* genes, and low level of *WRKY* TFs family. *SnRK2.2* is discussed above. *MDH* encodes malate dehydrogenase, this enzyme is critical in malate metabolism and is related in ROS producing genes (Akbar et al., 2020). *WRKY* TF family are associated to transduction of stress signaling and play a major role in plant defense to abiotic and biotic stress (Li et al., 2020c).

The effect of humic acid in foxtail millet plants under drought conditions (natural simulation conditions) was studied (Shen et al., 2020). Transcriptome sequencing and RT-qPCR was performed on plants. Humic acid caused a significant increase in the yield, dry weight and root-shoot ratio. *SETIT_021707mg*, *SETIT_016840mg*, and *SETIT_ 015030mg* genes were significantly up-regulated, while *SETIT_004913mg* and *SETIT_016654mg* genes were significantly down-regulated in the plants treated with humic acid and drought. These genes are related with metabolic pathways, secondary metabolite biosynthesis and starch and sucrose metabolism.

The effect of fulvic acid in tea plants (*Camellia sinensis*) under drought stress was studied (Sun et al., 2020). The authors examined the transcriptomics and metabolomics profiles, 604 and 3331 differentially expressed metabolite genes (DEGs) were found in plants at 4 and 8 days under drought respectively. DEGs are related in diverse biological processes such as ascorbate metabolism (*GME*, *AO*, *ALDH*), glutathione metabolism (*GST*, *G6PDH*, *CYS-GYL*), and flavonoids biosynthesis (*C4H*, *CHS*, *F3′5′H*, *F3H*). Ascorbic acid functions as an enzymatic cofactor and antioxidant plays roles in maintenance of ROS homeostasis (Conklin and Barth, 2004). Glutathione is one of the important endogenous antioxidants in plants, which functions as a substrate in antioxidative defense mechanisms by scavenging free radicals, conjugating to toxic electrophilic compounds, and reducing peroxides (Anderson and Davis, 2004). Flavonoids are polyphenol compounds with antioxidant activities, the accumulation of flavonoids could be a key step in development of plant tolerance to different stresses (Wang P. et al., 2018).

Using *Paeonia ostii* plants under natural drought stress (it was mainly characterized by low soil water content, and the roots of plants cannot absorb enough water to compensate for the consumption of transpiration) the effect of fulvic acid was studied (Fang et al., 2020). The fulvic acid treatment increased the leaf water content and antioxidant enzyme activities and decrease proline content, ROS accumulation, and relative electrical conductivity. Also, increased the expression level of drought-tolerant genes, like *CCoAOMT*, *CAB37*, *AGT1*, *At4g26520*. *CCoAOMT* ecodes caffeoyl-coenzyme A O-methyltransferase, this enzyme is involved in monolignol synthesis that affects the efficiency of lignification and lignin composition (Rakoczy et al., 2018), and the changes of lignin composition may serve in stress resistance. *CAB37* is a important regulatory site of photosynthesis under drought stress (Li et al., 2020d). *AGT1* is related to photosynthetic processing and photorespiration (Fang et al., 2020). *At4g26520* is ABA dependent signaling pathway in drought response (Fang et al., 2020).

Examination of Table 5 allows determining the absence of transcriptomic responses that were common among the six studies of humic and fulvic acids. One possible explanation, in addition to the small number of studies available, is the presence of chemical or physicochemical differences between the humic substances, or the fact that not all the studies were carried out under the same type of stress.

## Chitosan and Other Biopolymers

Chitosan is a polymer obtained by the deacetylation of chitin extracted from crustaceans, fungi, and insects. Chitosan is composed of N-acetyl-D-glucosamine and D-glucosamine units that have different degrees of deacetylation (Riaz Rajoka et al., 2020). The physiological effects of chitosan in plants are related to the ability of this polycationic compound to bind to a wide range of cellular components, such as the plasma membrane, cell wall components, and DNA, in addition to binding to specific receptors involved in plant defense (Katiyar et al., 2015). Other natural and synthetic polymers can be used in agriculture, including polyacrylates, polyacrylamides, and polysaccharides (Ekebafe et al., 2011).

Genes and their function are described below on the basis of the results of five studies in which chitosan-based biostimulants and other biopolymers were applied to plants under abiotic stress (Table 5).

Using tomato plants, researches (Hernández-Hernández et al., 2018) studied the application of hydrogels of nCu-chitosan-PVA and chitosan-PVA as promoters of tolerance to salt stress (100 mM NaCl). The treated and stressed plants presented improved growth and increased expression of *JA* and *SOD* genes at 48 h after stress. The *JA* gene was related to the biosynthesis of jasmonic acid, which has been shown to improve tolerance to osmotic and oxidative stress in plants under salinity stress (Zhao et al., 2014). *SOD* is discussed above.

Using maize plants under salinity stress (100 mM NaCl), researchers (Turk, 2019) studied the ability of foliar applications of chitosan (0.1%) to mitigate this stress. The treated plants presented increased growth and higher expression of the *AOX1* gene on the third day after the treatment; the *AOX1* gene encodes the mitochondrial alternative oxidase enzyme. This enzyme is involved in the alternative dissipative flow of the electron transport chain, in addition to optimizing the metabolism of respiration under normal and stress conditions (Erdal and Turk, 2016). *AOX1* also plays an essential role in modulating the balance between carbon and nitrogen (Hu et al., 2017).

Using *A*. *thaliana* seedlings grown *in vitro* under salinity stress (100 mM NaCl), researchers (González-Pérez et al., 2018) studied the effect of a biostimulant based on xyloglucan oligosaccharides extracted from *Tamarindus indica* L. (0.1 mg L^–1^) applied to the growth media. An increase in the expression of the *CAT3* gene was reported 24 h after stress, and the *CAT* gene is discussed above.

The effect of chitosan oligosaccharide (COS) on tea plants (*C. sinensis*) under cold stress (−4°C) was studied (Li et al., 2020e). The activity of SOD and POD, content of soluble sugar and chlorophyll in COS-treated tea plant were increased. The tea plants also were analyzed by transcriptomics with RNA-sequencing. There were identified 4503 DEGs between the control and COS under cold stress. By RT-qPCR, the GH3 gene expression was significantly higher in COS-treated plants (under stress). *GH3* (indole-3-acetic acid) is an important response gene of auxin-responsive protein, encode a class of IAA-amido synthetase related for balancing endogenous free IAA content, and play an important role in plant growth and development (Feng et al., 2015).

Using soybean under salt-stress, the effect of chitosan modified biochar (CMB) was studied (Mehmood et al., 2020). CMB treatment with salt-stress increased plant growth, root architecture characteristics, biomass yield, nutrients acquisition, chlorophyll, soluble protein and sugar contents. The gene expression levels triggered by salinity but with the application of CMB significantly increased the expression profile of *CAT*, *APX*, *POD*, *SOD*, *GmSALT3*, and *CHS* genes. *CAT*, *APX*, *POD*, and *SOD* encodes antioxidant enzyme and are discussed above. *GmSALT3* (salt tolerance-associated gene on chromosome 3) is associated with limiting the accumulation of sodium ions in shoots and a substantial enhancement in salt tolerance in soybean (Guan et al., 2014). *CHS* encode a chalcone synthase, this enzyme is the first key enzyme in the biosynthesis of flavonoids (El-Esawi et al., 2018a).

The review of Table 5 allows determining the absence of transcriptomic responses that were common among the five studies of chitosan and other biopolymers, except for the expression of antioxidant enzymes. More studies are needed with the same chitosan and others biopolymers in different species of plants, under the same stress condition.

## Inorganic Compounds

Beneficial elements are chemical elements that are not essential for all plants and are related to growth promotion (Pilon-Smits et al., 2009). The main beneficial elements include Si, Se, Co, Al, and Na. These are present in soils and in plants as different inorganic salts and as insoluble forms (Jardin, 2015).

The following describes the genes and their function obtained from the results of six studies in which biostimulants based on inorganic compounds and mixtures with other types of biostimulants were applied to plants under abiotic stress (Table 6).

**TABLE 6:** Description of genes whose transcript level changed in response to the application of biostimulants based on inorganic compounds and mixtures to plants under abiotic stress.

**Gene/gene expression time**	**Biostimulant type**	**Abiotic stress type**	**Crop/gene expression organ**	**References**
*OsDREB2A*, *OsNAC5*, *OsRDCP1*, *OsCMO*, *OsRab16b*/not specified	Selenium and silicon	Drought	Rice/seedlings	Khattab et al., 2014
*SlPIP1;5*, *SlPIP2;6*/3 and 5 days after treatment and stress	Silicon	Drought	Tomato/roots	Shi et al., 2016
*SbPIP1;3/1;4*, *SbPIP1;6*, *SbPIP2;2*, *SbPIP2;6*, *SbPIP2;3*/4 and 24 h after stress	Silicon	Drought	Sorghum/roots	Liu P. et al., 2014
*MT2a*, *MT2b*, *PCS1*, *CSD1*, *CSD2*/3 days after treatment	Silicon	Copper toxicity	*Arabidopsis/*leaves	Khandekar and Leisner, 2011
*Os08g02630 (PsbY)*, *Os05g48630 (PsaH)*, *Os07g37030 (PetC)*, *Os03g57120 (PetH)*, *Os09g26810*, *Os04g38410*/72 h after treatment	Silicon	Zinc toxicity	Rice/leaves	Song et al., 2014
*RAB18*, *P5CS1*, *ERF1A*, *MYB75*/field capacity 0.1 (10%)	Three different biostimulants (*A*. *nodosum* extract, amino acids, and potassium phosphite)	Drought	*Arabidopsis/*leaves	Fleming et al., 2019

Using two rice cultivars under drought stress (no irrigation for 10 days), researchers (Khattab et al., 2014) studied the application of sodium selenate (0.03 mM) and potassium silicate (1.5 mM) to the seeds to promote stress tolerance. Increased expression of genes such as *DREB2A*, *NAC5*, *RDCP1*, *CMO*, and *RAB16b* was reported. *DREB2A* and *NAC5* genes encode TFs. *NAC5* TFs activate genes related to the production of osmolytes, redox homeostasis, detoxification, and formation of macromolecules (Hu et al., 2008). *RDCP1* participates in a set of physiological responses to counteract dehydration stress (Bae et al., 2011). *CMO* encodes a choline monooxygenase that participates in glycine betaine biosynthesis, resulting in increased tolerance to salinity stress (Luo et al., 2012). *RAB16B* encodes the dehydrin-like LEA protein, whose expression which is induced in response to abiotic stress, and this protein is ABA dependent (Bies-Ethève et al., 2008).

Using tomato plants under drought stress (induced with 10% polyethylene glycol-6,000 added to the irrigation solution), researchers (Shi et al., 2016) studied the effect of potassium silicate (2.5 mM) added to the irrigation solution. The results showed that Si improved the growth, photosynthesis, and water status of plants under stress. The expression of the *SIPIP1;5* and *SIPIP2;6* genes increased at 5 and 6 days, respectively. Both genes are discussed above.

Using sorghum plants, researchers (Liu P. et al., 2014) studied the effect of applying sodium silicate (1.67 mM) to counteract water-deficit stress (10% PEG-6000). Increased expression of *SbPIP1;3/1;4*, *SbPIP1;6*, *SbPIP2;2*, *SbPIP2;6*, and *SbPIP2;3* was reported at 4 and 24 h after stress, and this group of genes encodes aquaporins and is discussed above.

Using *A*. *thaliana* plants under stress caused by copper toxicity (30 μM), researchers (Khandekar and Leisner, 2011) evaluated the effect of potassium metasilicate (1.5 mM). The *MT2a*, *MT2b*, *PCS1*, *CSD1*, and *CSD2* genes increased at 3 days after treatment. The *MT2a* and *MT2b* genes encode metallothioneins, which are proteins that bind to Cu to regulate its concentration in the cell (Guo et al., 2008). *PCS1* encodes a phytochelatin, which, like metallothioneins, binds to heavy metals and transports them to the vacuole (Adams et al., 2011). *CSD1* and *CSD2* encode the Cu/Zn SOD enzymes and are discussed above.

Using rice plants, researchers (Song et al., 2014) studied the effect of silicic acid (1.5 mM) added to the irrigation solution to increase stress tolerance caused by Zn toxicity (2 mM irrigation solution). The levels of the genes *Os08g02630 (PsbY)*, *Os05g48630 (PsaH)*, *Os07g37030 (PetC)*, *Os03g57120 (PetH)*, *Os09g26810*, and *Os04g38410* increased in response to the application of Si and stress at 72 h after the start of the treatments. The *Os08g02630 (PsbY)* gene encodes a photosystem II polyprotein, which, when its expression increases, subsequently increases both the activity of photosystem II and the amount of chlorophyll (Von Sydow et al., 2016). *Os05g48630 (PsaH)* encodes a photosystem I subunit (Zhang and Scheller, 2004), and *Os07g37030 (PetC)* encodes a polypeptide that binds to the Rieske FeS center of cytochrome bf and regulates the electron transport of the b_6_f complex during photosynthesis (C Breyton et al., 1994). *Os03g57120 (PetH)* encodes a ferredoxin-NADP^+^ reductase, which regulates the level of reduced glutathione in the cell (Song et al., 2014).

Using *Arabidopsis* plants under drought stress, researchers (Fleming et al., 2019) evaluated three commercial products based on *A*. *nodosum*, amino acids, and potassium phosphite. An increase in the expression of the *RAB18*, *P5CS1*, *ERF1A*, and *MYB75* genes was reported in response to the application of the products and at a field capacity of 0.1 (10%). The *ERF1A* gene encodes a TF of the ERF (ethylene response factor) family, with ethylene being a highly important hormone in plants (Trujillo-Moya and Gisbert, 2012). The other genes are discussed above.

Examination of Table 6 allows determining a few transcriptomic responses that were common among the six studies. Common genes in some studies were related to TFs, antioxidants and aquaporins. More studies are needed with the same inorganic compound in different species of plants, under the same stress condition.

**FIGURE 1:**
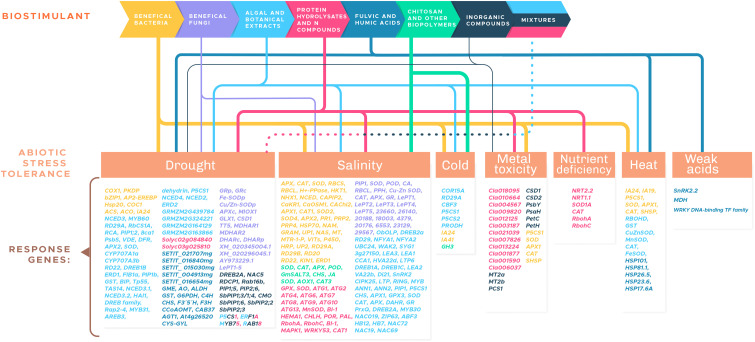
Global view of the differentially expressed genes in plants treated with different types of biostimulants that are involved in the tolerance of various types of abiotic stress.

As a summary of the collection of all the genes differentially expressed in response to the application of biostimulants to plants under abiotic stress, Figure 1 shows an outlook where the different types of biostimulants are presented according to the categorization described in du Jardin (2015). In addition to the 7 categories, mixtures based on 2 or more different types of biostimulants were added. Subsequently, the 7 different types of abiotic stress described in this review are shown. Finally, under each type of stress, DEGs are listed that may be part of the transcriptional modification of plants and may be involved in abiotic stress tolerance. Biostimulants based on algal and botanical extracts have been extensively studied in plants under different types of abiotic stress, such as drought, salinity, cold and heat. On the other hand, biostimulants based on protein hydrolysates and compounds with N have been studied in response to only various types of abiotic stress, such as drought, salinity, metal toxicity, and nutrient deficiency. The most studied is drought stress, followed by salinity, metal toxicity, low and high temperature, weak acids, and nutrient deficiency. However, considering that high-temperature stress (in the form of heatwaves) has become increasingly common in tropical and subtropical regions as climate change progresses, it is necessary to carry out additional studies to verify the usefulness of different biostimulants under extreme temperature conditions.

**FIGURE 2:**
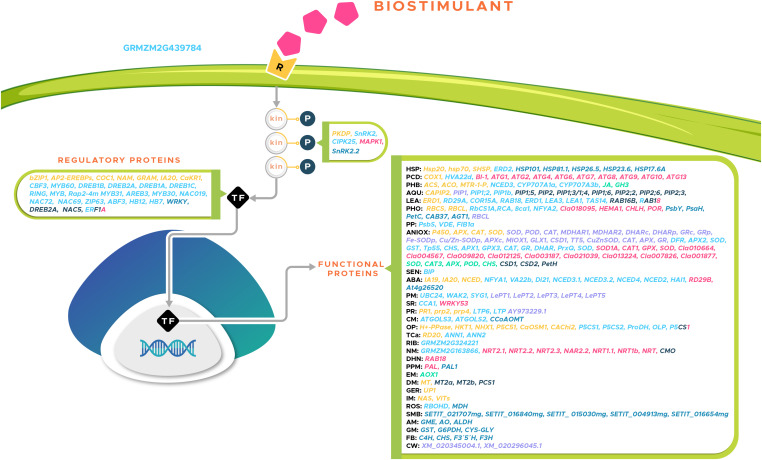
Cellular representation of the functional categories of genes differentially expressed in plants in response to the application of different types of biostimulants. R: receptor, kin: kinases, P: phosphate groups, TF: transcription factor, HSP: heat-shock protein, PCD: programmed cell death, PHB: phytohormone biosynthesis, AQU: aquaporin, LEA: late embryogenesis abundant, PHO: photosynthesis, PP: photoprotection, ANTIOX: antioxidant metabolism, SEN: senescence, ABA: biosynthesis and signaling of ABA, FOS: phosphorous metabolism, SR: stomatal regulation, PR: PR protein, CM: carbohydrate metabolism, OP: osmoprotectant, TCa: CaC2 transport, RIB: ribosome, NM: nitrogen metabolism, DHN: dehydrin, PPM: phenylpropanoid metabolism, EM: energy metabolism, DM: metal detoxification, GER: germination, IM: iron metabolism, ROS: reactive oxygen species, SMB: secondary metabolite biosynthesis, AM: ascorbate metabolism, GM: glutathione metabolism, FB: flavonoids metabolism, CW: cell wall. Gene colors: beneficial bacteria, beneficial fungi, algal and botanical extracts, protein hydrolysates and N compounds, fulvic acids and humic acids, chitosan and other biopolymers, inorganic compounds, biostimulant mixtures.

In the present review, emphasis was placed on presenting DEGs in plants treated with biostimulants, as well as presenting the cellular function of these genes to try to decipher the transcriptional mechanism underlying the tolerance to abiotic stress. Figure 2 depicts how biostimulants are perceived in cells through receptors. Such recognition triggers a series of downstream events where kinase-like proteins and TFs (regulatory proteins) drive signal transduction, after which specific response genes are ultimately activated to produce functional proteins to counteract the effects caused by abiotic stress. In the genes presented in Figure 2, overlap in the functional categories of functional-type proteins can be observed, in which some genes are homologous since they have the same function in different plant species. Some orthologous genes are also presented in different plant species with similar functions due to a common ancestor. However, to support this information, it is necessary to carry out a phylogenetic analysis of the sequences. Among the functional categories that present the most DEGs are proteins with a role in antioxidant metabolism, followed by functions involving photosynthesis, biosynthesis and signaling of ABA, PCD, aquaporins, osmoprotectants, LEA-like proteins, nitrogen metabolism, heat-shock proteins, biosynthesis of phytohormones, phosphorus metabolism, PR proteins, secondary metabolite biosynthesis, metal detoxification, flavonoids metabolism, photoprotection, carbohydrate metabolism, Ca^+2^ transport, ascorbate metabolism, glutathione metabolism, stomatal regulation, metabolism of phenylpropanoids, iron metabolism, ROS, cell wall, ribosomes, dehydrins, energy metabolism, senescence, and germination. It is difficult to know if the abovementioned categories correspond to the spectrum of biological responses induced by biostimulants or if the spectrum is an effect of the sampling of the works used in this study (a result of the choice of plant species, type of stress, and the genes chosen in the different works). More studies of specific metabolic pathways that represent plant status against stresses, for example, antioxidant metabolism, biosynthesis and signaling of ABA, PCD, photosynthesis, water transport, and C or N metabolism, are needed. A massive study of gene expression is also proposed through the analysis of transcriptomes or complete proteomes.

With the information analyzed in this review, in Table 7 some putative molecular markers are proposed that can be used to test the potential of different types of biostimulants under different stress conditions. The genes selected as molecular markers are functional proteins that could be associated with tolerance to abiotic stress in plants.

**TABLE 7:** Putative molecular markers for different potential biostimulants in varying abiotic stress conditions.

**Molecular markers**	**Function**	**Biostimulant type**	**Abiotic stress type**
CAT, SOD, POD, APX, GPX, GR, CHS, CSD1, DHAR	Antioxidant metabolism	Beneficial bacteria, beneficial fungi, algal and botanical extracts, protein hydrolysates and N compounds, chitosan and other biopolymers, inorganic compounds	Drought, salinity, metal toxicity, nutrient deficiency, heat
HSP70	Heat-shock protein (HSP)	Beneficial bacteria, algal and botanical extracts	Drought, salinity
PIP1, PIP2	Aquaporin	Beneficial bacteria, beneficial fungi, algal and botanical extracts, inorganic compounds	Drought, salinity
ERD1, RAB18	Late embryogenesis abundant (LEA)	Beneficial bacteria, algal and botanical extracts, mixtures of biostimulants	Drought, salinity
NCED	Biosynthesis and signaling of ABA	Beneficial bacteria, algal and botanical extracts	Drought, salinity
P5CS1	Osmoprotectant	Beneficial bacteria, algal and botanical extracts, mixtures of biostimulants	Drought, salinity, cold, metal toxicity, heat
PAL	Phenylalanine ammonium lyase (PAL)	Protein hydrolysates and N compounds, fulvic and humic acids	Salinity

## Conclusion and Perspectives

Given the information described above, genes that can act as molecular markers in abiotic stress tolerance can be globally visualized by applying biostimulants. The genes described encode functional proteins as well as regulatory proteins.

Most related studies have focused on the use of algal extracts, especially those of *A. nodosum*, and the effects of drought and salinity stress have been the most explored. Thus, there is an area of opportunity to study the different types of biostimulants and abiotic stresses that have been studied little.

However, to focus efforts of future research on the analysis of DEGs in plants in response to the application of biostimulants (those with greater accessibility to producers such as humic substances, vegetable extracts, hydrolysates, and elements such as silicon), it is necessary to take into account several factors, such as the type of biological model (study species with high nutritional importance, such as potato, wheat, maize, rice and tomato), the class of biostimulant, the concentration of the biostimulant (focus on commercial recommendations), the type of application (depending on the crop species and the type of biostimulant), the study organ (include leaves and, if possible, the organ of commercial interest, such as the roots, tubers and fruits), and the time of analysis of gene expression (establish comparable standards of sampling times at 12, 24, and 36 h after the application of the biostimulant).

## Author Contributions

AB-M: conceptualization. SG-M: writing of the original draft and editing. SG-M, SS-G, MV-C, AJ-M, AL-T, and AB-M: responsible for processing and organizing information. All authors have read and approved the final manuscript.

## Conflict of Interest

SS-G and MV-C were employed by company UPL Mexico. The remaining authors declare that the research was conducted in the absence of any commercial or financial relationships that could be construed as a potential conflict of interest.
